# Good practices in bilingual children

**DOI:** 10.1186/1824-7288-40-S1-A80

**Published:** 2014-08-11

**Authors:** Francesca Cucinotta, Roberta Maggio, Matilde Gagliano, Maria Venuti, Antonella Gagliano

**Affiliations:** 1 UOC di Neuropsichiatria Infantile, Policlinico Universitario G. Martino, Messina, Italy

## 

Bilingualism is the regular use of more than one language in everyday life [1,2]. In our country, 47.2% of students were born from non-Italian parents [3], and in Europe, bilingualism is even more prevalent: 56% of the population across all European Union countries is reported being functionally bilingual [4]. So it is critical to understand risks and protective factors specific to the development of bilingual children for clinical and educational reasons.

It is true that linguistic tasks are often performed more poorly by bilingual children than monolinguals [5,6], especially as regards assessment of vocabulary [7,8], picture-naming tasks [9,10], comprehending and producing words [11,12]. It is also common among bilingual children who learn Italian as a second language, to have lower skill levels than those of monolinguals, in relation to reading and writing or understanding of texts [13]; but these results cannot be described as a pathological outcome.

In contrast to this pattern, bilinguals at all ages demonstrate better executive control than monolinguals matched in age and other background factors.

In the last decades, many studies showed that the experience of early exposure to two languages, and the constant practice of selecting the target language avoiding intrusions of the non-target language, can improve skills such as selective attention, inhibition and cognitive control with respect to non-verbal tasks. Other benefits also include the early development of metalinguistic ability and better achievements in working memory tasks.

These effects have been found at all stages across the life span, beginning from infancy and toddlerhood, continuing through young adulthood and up to older age [14-17].

Thus, it is a matter of considerable concern with the large and growing dual language population, how to properly recognize normal and abnormal dual language development, and several important implications can be derived from extant developmental and clinical research: a language disorder should be suspected in a dual language child, when the child is reported to be significantly behind in the understanding of both languages, although there has been significant exposure to both languages, and when there are language-based learning problems [18]. While it has been clearly documented that bilingualism does not cause language delay or language disorders [19], the latter are certainly possible in bilingual children. Such possibility should not be easily dismissed and slight delays should not instead be misattributed to the child’s bilingual condition even though a more severe delay must be assessed.
